# Self-Assembly of Antibacterial Polymer Nanotubes with Chlorine Regenerability

**DOI:** 10.3390/biom16050725

**Published:** 2026-05-14

**Authors:** Shina Mao, Zhizhan Ji, Xu Yang, Jiayu Li, Haoran Gao, Il Kim, Yu Zhang

**Affiliations:** 1 Digital and Intelligent Empowerment Biomedical Innovation Center, School of Pharmacy, Shanghai University of Medicine and Health Sciences, Shanghai 201318, China; 2 School of Chemical Engineering, Pusan National University, Busan 46241, Republic of Korea

**Keywords:** hyper-crosslinked polymers, antibacterial, *N*-halamines, 5,5-dimethylhydantoin

## Abstract

Bacteria pose significant threats to human health, industrial production, and daily life, with widespread microbial contamination remaining a critical challenge for global public health. Conventional porous materials often suffer from insufficient antibacterial efficacy, necessitating the development of advanced antimicrobial systems. Herein, we report a synthetic strategy for fabricating chloride-regenerable porous tubular polymers (HCP-DMH-Cl) via a combination of Friedel–Crafts alkylation and nucleophilic substitution reactions. HCP was initially prepared through a crosslinking reaction via Friedel–Crafts alkylation using FeCl_3_ as the catalyst and benzyl alcohol as the monomer. SEM characterization was performed to validate the tubular architectural morphology of HCP. The polymeric *N*-halamine precursor, HCP-DMH, was subsequently obtained through stepwise bromomethylation and nucleophilic substitution modifications. Upon chlorination, HCP-DMH-Cl exhibited good antibacterial efficacy against both *E. coli* and *S. aureus*, coupled with favorable regenerability of its oxidative chlorine content. This approach paves the way for designing next-generation porous media with tailored antibacterial functionality and sustainable chlorine-release capabilities.

## 1. Introduction

In recent years, bacterial infections have profoundly impacted human daily life. Notably, among diverse bacterial strains, *Staphylococcus aureus* (*S. aureus*) and *Escherichia coli* (*E. coli*) emerge as primary pathogens requiring urgent attention [1,2,3]. These bacteria are distinguished by their remarkable capacity for dissemination through multiple transmission routes. Infections caused by these microorganisms can elicit a spectrum of diseases, including skin infections, respiratory tract infections, osteomyelitis, and bacteremia [4,5]. Water pollution serves as a critical reservoir for bacterial proliferation, with microbes entering water bodies via soil particle migration [6,7,8]. Pathogenic microorganisms (e.g., *Salmonella*, *Shigella*) often adhere to sediments and spread through runoff, forming persistent pollution sources [9,10,11]. Untreated animal feces, carrying pathogens like *E. coli*, further contaminate water systems, particularly in rural areas, where livestock farming contributes to a 42% percolation pollution rate [12,13]. Additionally, agricultural fertilizers exacerbate soil erosion, while pesticide residues combine with bacteria to form composite pollutants, escalating toxic impacts on aquatic ecosystems [14,15,16,17]. Consequently, effective environmental disinfection strategies for polluted water bodies are urgently required.

The practical application of many antimicrobial materials is restricted by inherent limitations, such as residue risks from metal-based agents, limited stability of antimicrobial peptides, and the operational inflexibility of photocatalytic materials [18,19]. Additionally, issues like high production costs and poor scalability further impede their widespread adoption [20]. Porous organic polymers (POPs) are functional materials featuring permanent pore structures, constructed from lightweight elements (C, H, O, N, etc.) linked by covalent bonds [21,22,23]. Hyper-crosslinked polymers (HCPs), a representative class of POPs, have emerged as one of the most commercially promising materials due to their low cost and lightweight architecture [24,25]. HCPs are synthesized via crosslinking polymerization of various monomers, often facilitated by Friedel–Crafts reactions [26,27]. However, the rapid kinetics of these reactions typically yield HCPs with non-uniform morphologies dominated by microporous structures. Numerous studies have confirmed the critical role of HCPs in adsorptive removal of pollutants, including dyes, heavy metals, and organic solvents [28,29,30]. Nevertheless, environmental disinfection remains a pressing challenge, particularly in the context of bacterial contamination, one of the most prevalent forms of water pollution, rife in sources contaminated by sewage, fecal matter, or agricultural runoff [31,32,33]. To address this, there is an urgent need for porous materials with hierarchical pore systems, enabling dual functionality for pollutant adsorption and antibacterial regenerability.

A key limitation of traditional HCPs lies in the rapid kinetics of Friedel–Crafts reactions, which typically yield irregular morphologies that hinder mass transfer efficiency [34,35]. Our lab has proposed a Lewis acid-base templating strategy to precisely control HCPs architecture [36,37,38,39]. By modulating catalyst ratios and reaction conditions, this approach enables the facile one-pot synthesis of uniform tubular morphologies. Surface functionalization with reactive pendant groups further facilitates covalent grafting of antibacterial agents, enhancing biocidal efficacy and regeneration capacity.

**Figure 1:**
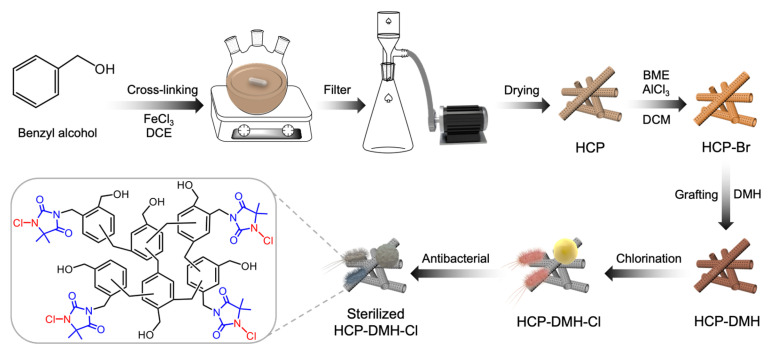
A schematic diagram is presented to illustrate the preparation process of HCP-DMH-Cl.

In this study, we describe a synthetic approach for preparing chloride-regenerable porous tubular antibacterial polymers via a combination of Friedel–Crafts alkylation and nucleophilic substitution reactions. As depicted in Figure 1, the tubular morphology of HCP was readily achieved through a one-pot Friedel–Crafts crosslinking reaction catalyzed by FeCl_3_, leveraging benzyl alcohol as the monomeric building block. Subsequently, HCP-Br was synthesized by alkylating the aromatic moieties of HCP using bromomethyl methyl ether (BME) in the presence of AlCl_3_. The polymeric *N*-halamine precursor, HCP-DMH, was then obtained via nucleophilic substitution between HCP-Br and 5,5-dimethylhydantoin (DMH). Upon chlorination with sodium hypochlorite solution, HCP-DMH was converted to its biocidal form, HCP-DMH-Cl. Antimicrobial assays confirmed that HCP-DMH-Cl efficiently inactivates both *S. aureus* and *E. coli*. This protocol offers valuable insights for designing porous materials with enhanced antibacterial regenerative capacity, holding promise for environmental remediation applications.

## 2. Materials and Methods

### 2.1. Materials

Benzyl alcohol, 1,2-dichloroethane (DCE), dichloromethane (DCM), DMH, and BME were obtained from TCI (Shanghai, China). Potassium hydroxide (KOH), sodium hypochlorite (NaClO), psulfuric acid (H_2_SO_4_), sodium thiosulfate (Na_2_S_3_O_3_), potassium iodide (KI, 99%), anhydrous ferric chloride (FeCl_3_, 98%), aluminum chloride (AlCl_3_, 98%), ethanol (99%), and phosphate-buffered saline (PBS) were purchased from Adamas-Beta (Shanghai, China).

### 2.2. Methods

Fourier transform infrared (FT-IR) spectra were recorded on a Nicolet iS50 spectrometer (Thermo Fisher Scientific, Waltham, MA, USA) to analyze the chemical structure of the synthesized polymers. The samples were prepared as KBr pellets and measured in the wavenumber range of 4000–400 cm^−1^ with a resolution of 4 cm^−1^. X-ray photoelectron spectroscopy (XPS) analysis was performed using a Theta Probe angle-resolved system (Thermo Fisher Scientific, Waltham, MA, USA) equipped with monochromatic Al *K*_α_ radiation, providing an energy resolution of 0.4 eV for high-resolution spectra. Surface morphologies were examined by scanning electron microscopy (SEM) using a Quanta FEG 250 instrument (FEI Company, Hillsboro, OR, USA). Prior to observation, samples were fixed onto aluminum stubs with conductive carbon tape and coated with a thin gold layer. Water contact angle (WCA) measurements were conducted on a DSA30 system (KRÜSS GmbH, Hamburg, Germany) via the sessile drop method, using 5 μL deionized water droplets, and the reported values represent the average of five measurements. X-ray diffraction (XRD) patterns were collected on a D8 Advance diffractometer (Bruker, Karlsruhe, Germany) with Cu *K*_α_ radiation over a 2*θ* range of 5–80° at a scanning rate of 5° min^−1^. All data analysis and graph plotting in this study were performed using Origin software (Version 2023b, OriginLab Corporation, Northampton, MA, USA).

### 2.3. Synthesis of HCP

HCP was synthesized following previously reported protocols [36,37,38,39]. In a typical synthesis, 138.2 mg of benzyl alcohol was dissolved in 100 mL of DCE within a Schlenk flask. Subsequently, 324.4 mg of FeCl_3_ was added, and the reaction was conducted at 80 °C under a nitrogen atmosphere. The Friedel–Crafts hyper-crosslinking reaction proceeded for 18 h under moderate stirring. The reaction was quenched by adding a 4:1 ethanol/water solution, and the precipitate was washed sequentially with ethanol and acetone. The collected brown crude solid was transferred to a Soxhlet extractor and extracted with ethanol for 36 h to remove residual FeCl_3_ catalyst. The resultant HCP product was dried overnight in a vacuum oven at 60 °C.

### 2.4. Synthesis of HCP-Br

In a typical bromomethylation procedure, 50 mg of HCP was dispersed in 50 mL of DCM under magnetic stirring to form a homogeneous suspension. Following this, 0.5 mL of BME was added dropwise via a syringe, and 162.2 mg of anhydrous AlCl_3_ was introduced as the catalyst. The reaction mixture was refluxed at 40 °C for 8 h under a nitrogen atmosphere. Upon completion, the product was isolated by filtration, washed repeatedly with acetone, and dried overnight at 60 °C under vacuum.

### 2.5. Synthesis of HCP-DMH

HCP-DMH was synthesized following reported procedures [40]. DMH (0.2 g) and KOH (0.086 g) were first dissolved in 10 mL of deionized water in a 50 mL round-bottom flask under continuous stirring at 25 °C. After complete dissolution, ethanol (5 mL) and HCP-Br (0.1 g) were subsequently introduced. The reaction mixture was then refluxed at 60 °C for 6 h under magnetic stirring. Following the reaction, the system was allowed to cool naturally to room temperature, and the resulting solid was collected by vacuum filtration, rinsed with ethanol three times, and dried under vacuum at 60 °C.

### 2.6. Synthesis of HCP-DMH-Cl

HCP-DMH was chlorinated by immersion in a 10 wt% NaClO solution (pH 7) at 25 °C for 2 h. HCP-DMH-Cl was extensively rinsed with deionized water and dried in air. The content of active chlorine was quantified by iodometric titration. In a typical procedure, 0.05 g of the sample was dispersed in 15 mL of ethanol, followed by the addition of KI (0.1 g) and 0.04 N H_2_SO_4_ (3 mL). The suspension was stirred at 25 °C for 20 min to release iodine, which was subsequently titrated using 0.01 N Na_2_S_2_O_3_ solution in the presence of starch as an indicator. The oxidative chlorine content (ppm) was calculated using the formula:

Cl^+^ = *N* × *V* × 35.45 × 100/(2 × *W*) (1)

where Cl^+^ is the chlorine loading, *N* and *V* are the concentration and volume of Na_2_S_3_O_3_ solution, and *W* is the weight of HCP-DMH-Cl.

### 2.7. Antibacterial Activity Assay

The antibacterial activity of the HCP-DMH-Cl was evaluated using *E. coli* and *S. aureus* as model pathogens in a standard plate count assay. HCP-DMH-Cl was incubated with bacterial suspensions (1 × 10^6^ CFU/mL) at 37 °C. A 100 μL aliquot of the co-culture was serially diluted in PBS, and 10 μL of each dilution was spread onto nutrient agar- plates. The plates were incubated at 37 °C for 12 h, and antibacterial activity was quantified by counting viable colonies. All experiments were performed in triplicate. To assess the regenerability of HCP-DMH-Cl, 100 mL of 0.01 N Na_2_S_3_O_3_ solution was added to completely consume the active chlorine species. The material was then re-chlorinated using a 10 wt% NaClO solution as described in the chlorination protocol, restoring its antibacterial functionality.

## 3. Results and Discussion

### 3.1. Structural Evolution and Morphological Characteristics

**Figure 2:**
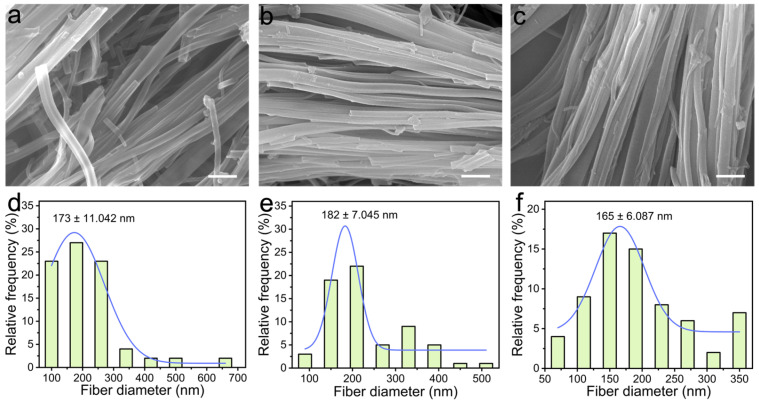
SEM images of (**a**) HCP, (**b**) HCP-Br, and (**c**) HCP-DMH. Scale bars: 1 μm. Corresponding size distribution profiles of the fibrous structures for (**d**) HCP, (**e**) HCP-Br, and (**f**) HCP-DMH.

SEM characterization systematically highlighted distinct morphological transitions across successive synthetic stages, providing critical insights into structure-property relationships. As shown in Figure 2a, the pristine HCP exhibited well-defined tubular architecture, featuring a relatively smooth surface interspersed with visible micropores consistent with literature reports on Friedel–Crafts-synthesized HCPs. This morphology aligns with the rapid crosslinking kinetics typical of Lewis acid-catalyzed polymerization, where microporosity arises from dense aromatic stacking [36,37]. Notably, bromination followed by DMH modification induced a marked increase in surface roughness and porosity, evidencing successful functional group grafting while preserving the porous framework (Figure 2b,c). Figure 2d–f display the corresponding size distribution profiles of fibrous structures, showing that HCP-DMH retained a tubular morphology despite surface modification, a critical indicator of synthetic consistency. This structural integrity ensured unimpeded mass transport to internal active sites. The abundance of nanopores is particularly significant, as it enhanced the specific surface area and created tortuous diffusion pathways, improving interaction kinetics with external entities (e.g., bacterial membranes or moisture) [41,42]. Such structural robustness and accessible porosity suggest potential utility, such as in reusable antibacterial surfaces. Compared with conventional dense beads or planar coatings, the tubular porous morphology may provide more exposed functional interfaces and reduced diffusion resistance for water and bacterial contact [43,44]. Unlike many surface-immobilized chlorinated coatings, where active sites are primarily confined to the outermost layer, the porous HCP framework enables broader spatial distribution of *N*-halamine groups throughout the accessible network [45,46]. This structural characteristic may contribute to the favorable antibacterial activity and regenerability observed in this study.

### 3.2. Characterization of Chemical Functional Groups

**Figure 3:**
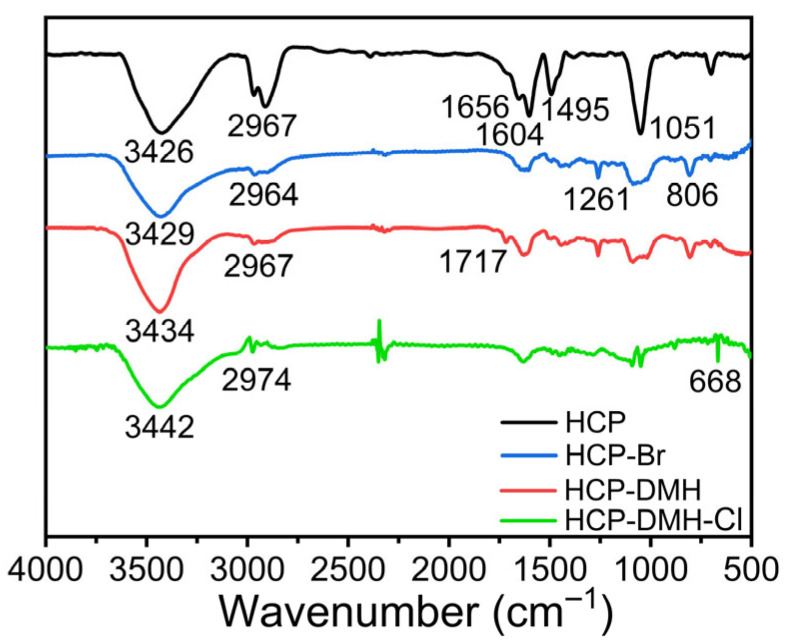
FT-IR spectra of HCP, HCP-Br, HCP-DMH, and HCP-DMH-Cl.

FT-IR spectroscopy and XPS analysis were conducted to confirm the chemical structure of these polymers. As illustrated in Figure 3, the FT-IR spectrum of pristine HCP exhibited characteristic absorption bands at 1656, 1604, and 1495 cm^−1^, assigned to the C–H stretching vibrations of the benzene ring and skeletal vibrations of aromatic frameworks, consistent with literature reports on Friedel–Crafts-synthesized HCPs [36,37,38,39]. The peak at 2967 cm^−1^ corresponded to symmetric stretching of methylene groups (–CH_2_–), while the broad band at 3426 cm^−1^ and sharp peak at 1051 cm^−1^ were attributed to C–O and O–H stretching vibrations, respectively, arising from residual hydroxyl groups and absorbed moisture. Upon bromination, new absorption peaks emerged at 1261 cm^−1^ (C–H deformation vibration in bromomethyl substituents) and 806 cm^−1^ (C–Br stretching vibration), confirming the successful introduction of bromine moieties. Notably, subsequent DMH modification induced a slight blue shift in these peaks, attributed to electron-donating effects from the dimethylhydrazine groups. Concomitantly, a new peak at 1717 cm^−1^ arose, assigned to the carbonyl stretching vibration of the hydantoin ring, providing direct evidence of DMH incorporation [40]. Chlorination resulted in global spectral modifications: all peaks exhibited reduced intensity and systematic blue shifts, consistent with inductive effects from electron-withdrawing chlorine atoms. Most significantly, a distinct absorption band appeared at 668 cm^−1^, unequivocally assigned to the N–Cl stretching vibration, a hallmark of biocidal *N*-halamine structures. The collective spectral transitions across synthesis stages provide robust spectroscopic evidence for the stepwise functionalization and final chlorination, substantiating the successful fabrication of the antimicrobial polymer HCP-DMH-Cl.

**Figure 4:**
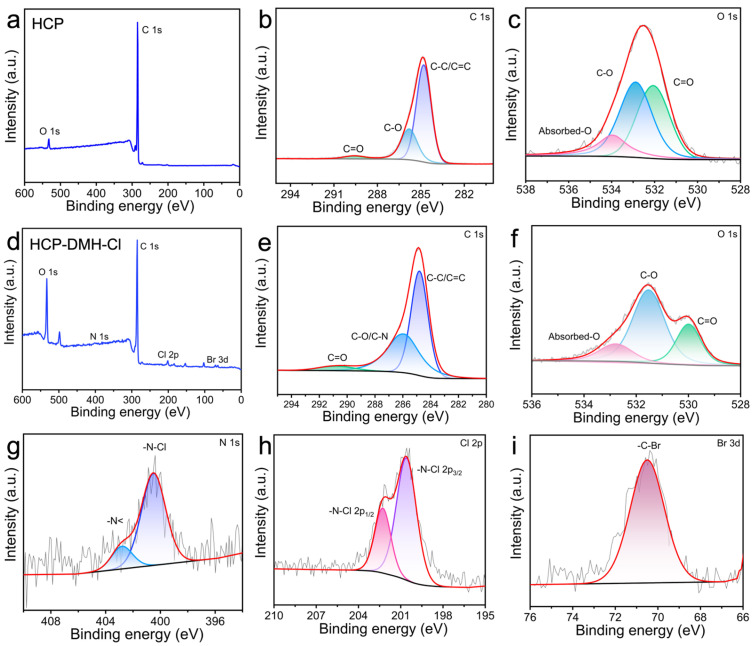
XPS characterization of the HCP and HCP-DMH-Cl. (**a**,**d**) XPS survey profiles of the HCP and HCP-DMH-Cl. Deconvoluted XPS profiles for (**b**,**e**) C 1s, (**c**,**f**) O 1s, (**g**) N 1s, (**h**) Cl 2p, and (**i**) Br 3d.

XPS analysis provided further evidence of the chemical transformations on HCP surfaces following nucleophilic substitution and chlorination. The pristine HCP exhibited characteristic core-level peaks at 287 eV (C 1s) and 532 eV (O 1s), while HCP-DMH-Cl displayed distinct new peaks at 401.3 eV (N 1s), 201 eV (Cl 2p), and 71.1 eV (Br 3d), as shown in Figure 4a,d. The appearance of N, Cl, and Br signals indicates that DMH groups were successfully introduced, followed by chlorination. Deconvolution of the C 1s spectrum for pristine HCP revealed three components: 284.7 eV (C–C/C=C), 285.8 eV (C–O), and 289.5 eV (C=O), consistent with the aromatic framework and residual oxygen-containing groups (Figure 4b) [36]. Chemical modification induced a significant broadening of the C–O peak (285.8 eV) due to spectral overlap with newly formed C–N bonds (286.5 eV), as evident in the deconvoluted profile of HCP-DMH-Cl (Figure 4e). This overlap indicates successful grafting of DMH, where nitrogen-containing functionalities contribute to the carbon binding environment. The O 1s spectra further corroborate structural changes: pristine HCP displayed peaks at 532.1 eV (C–O), 532.8 eV (C=O), and 533.9 eV (adsorbed oxygen) (Figure 4c). Following modification, the C=O peak shifted to 529.9 eV, a downfield shift attributed to the electron-withdrawing effect of the hydantoin carbonyl group in DMH (Figure 4f). This shift aligns with literature reports on *N*-halamine functionalized polymers, where carbonyl conjugation with nitrogen reduces oxygen electron density. The N 1s analysis of HCP-DMH-Cl unveiled two distinct components at 400.4 eV and 402.7 eV, assigned to imide nitrogen (–N<) in hydantoin rings and covalent N–Cl bonds, respectively (Figure 4g) [47]. The 2.3 eV separation between these peaks reflects the inductive effect of chlorine, which elevates the binding energy of N–Cl nitrogen relative to imide nitrogen. This assignment is corroborated by the Cl 2p spectrum, which exhibits a doublet at 200.6 eV (Cl 2p_3/2_) and 202.2 eV (Cl 2p_1/2_) with a 1.6 eV spin–orbit splitting, characteristic of covalent N–Cl bonds (Figure 4h) [48]. The presence of Br 3d signal in HCP-DMH-Cl (Figure 4i) further confirmed the intermediate bromination step, as Br is retained in unreacted sites prior to nucleophilic substitution. Collectively, these XPS findings provide a comprehensive picture of the synthetic pathway: from aromatic HCP to brominated intermediates, followed by DMH grafting and final chlorination. The spectral shifts and new peak assignments offer quantitative evidence of functional group transformations, aligning with FT-IR and SEM observations to validate the successful fabrication of antibacterial HCP-DMH-Cl.

### 3.3. Crystallinity and Hydrophilicity

**Figure 5:**
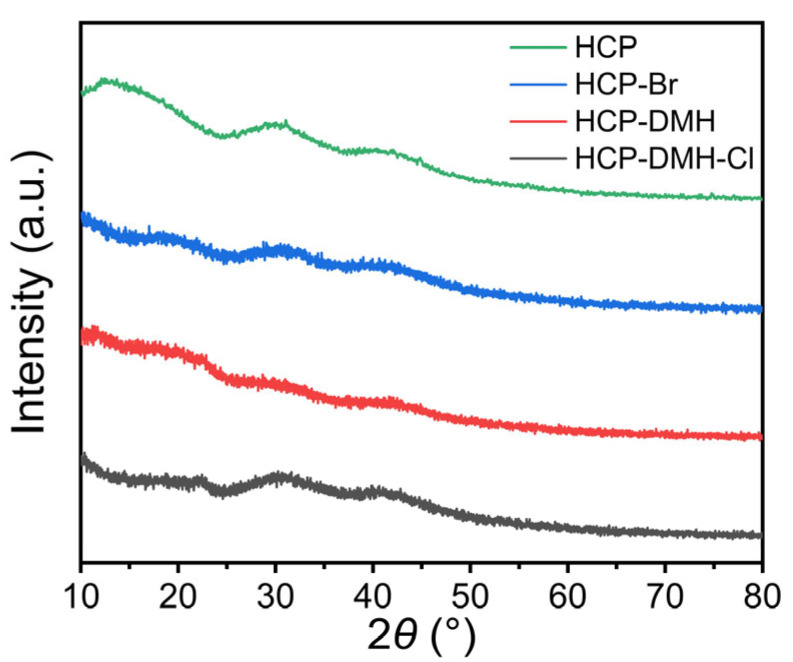
XRD spectra of the HCP, HCP-Br, HCP-DMH, and HCP-DMH-Cl.

The XRD patterns in Figure 5 exhibit two broad diffraction peaks at a 2*θ* range of 25–45°, characteristic of the amorphous carbonaceous matrix in HCPs. Notably, post-modification with DMH and chlorination did not alter the position or intensity of these peaks. This preservation indicates that the hierarchical porous architecture remains intact through the synthetic steps, a critical feature for maintaining adsorption capacity. For antibacterial applications, the amorphous nature facilitates intimate contact between N–Cl groups and microbial membranes.

**Figure 6:**
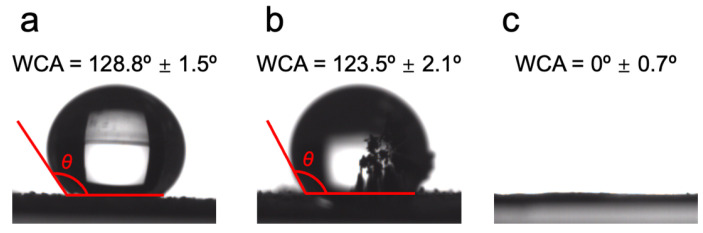
Water contact angle measurements of (**a**) HCP, (**b**) HCP-DMH, and (**c**) HCP-DMH-Cl.

WCA measurements revealed a pronounced hydrophilic transition in HCP-DMH-Cl relative to its precursors. Pristine HCP exhibited a WCA of 128.8° ± 1.5°, indicative of hydrophobic aromatic surfaces, while HCP-DMH maintained similar hydrophobicity (123.5° ± 2.1°) due to the non-polar nature of dimethylhydrazine moieties (Figure 6a,b). Notably, chlorination induced a dramatic decrease in WCA to 0° ± 0.7°, a significant reduction that aligns with the introduction of polar N–Cl bonds (Figure 6c) [49,50]. The enhanced hydrophilicity is of critical importance for biological applications, as it facilitates water absorption, improves dispersion in aqueous media, and promotes efficient diffusion of active chlorine species. These findings establish that chlorination-tuned hydrophilicity not only signifies successful N–Cl functionalization but also optimizes mass transport processes to enhance biocidal activity.

### 3.4. Oxidative Chlorine Content and Regenerability

**Figure 7:**
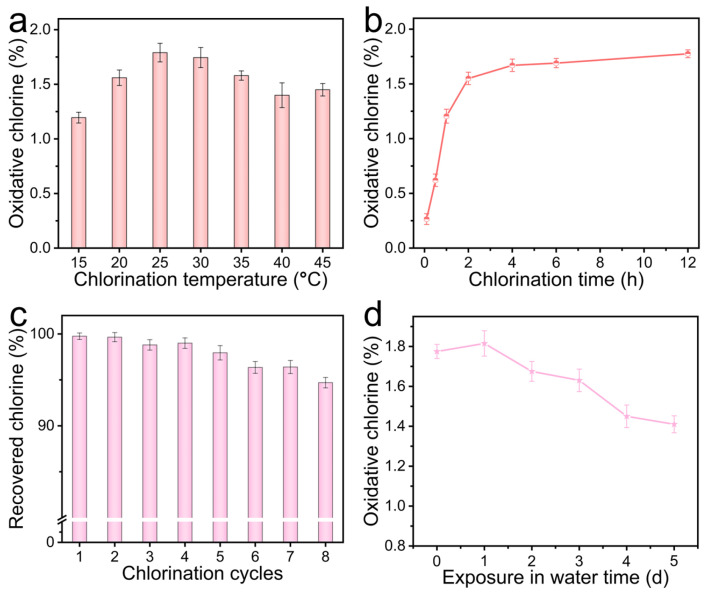
Influence of chlorination conditions, including (**a**) temperature and (**b**) duration, on the oxidative chlorine content of HCP-DMH-Cl. (**c**) Regenerability and (**d**) aqueous stability of HCP-DMH-Cl.

To elucidate the influence of chlorination conditions, the effects of temperature and duration on the HCP-DMH-Cl system were systematically investigated, given the critical role of oxidative chlorine content in antimicrobial performance. Figure 7a depicts the oxidative chlorine content as a function of chlorination temperature (15–45 °C) at a fixed duration of 2 h. A pronounced increase was observed upon raising the temperature from 15 to 25 °C, attributable to the acceleration of chlorination kinetics. Notably, between 25 and 30 °C, the oxidative chlorine content plateaued at 1.75%. Further temperature elevation to 45 °C resulted in a significant decline, consistent with thermally induced N–Cl bond cleavage and NaClO decomposition. Given the marginal difference between 25 and 30 °C, subsequent duration optimization was conducted at 25 °C. Figure 7b reveals that the oxidative chlorine content increased asymptotically with time, approaching saturation within 2 h. Prolonged chlorination beyond 2 h yielded negligible gains, reinforcing the efficiency of this protocol. Thus, it is evident that HCP-DMH-Cl synthesized at 25 °C for 2 h represents the optimal formulation, as it exhibits the highest oxidative chlorine content. The facile regenerability of *N*-halamines via simple re-chlorination represents a distinct advantage over traditional antimicrobial agents, as it ensures sustained biocidal efficacy. As shown in Figure 7c, HCP-DMH-Cl retained 95% of its initial oxidative chlorine content after eight chlorination-dechlorination cycles, demonstrating favorable structural resilience. In addition, the long-term aqueous stability of HCP-DMH-Cl was further evaluated to assess its durability under potential use-relevant conditions. As shown in Figure 7d, after immersion in water for extended periods, the oxidative chlorine content gradually decreased with increasing soaking time but remained above 1.4%. This result indicates that HCP-DMH-Cl possesses relatively good stability in aqueous environments, which is beneficial for sustained antibacterial performance in water-contact applications.

### 3.5. Antibacterial Activity

**Figure 8:**
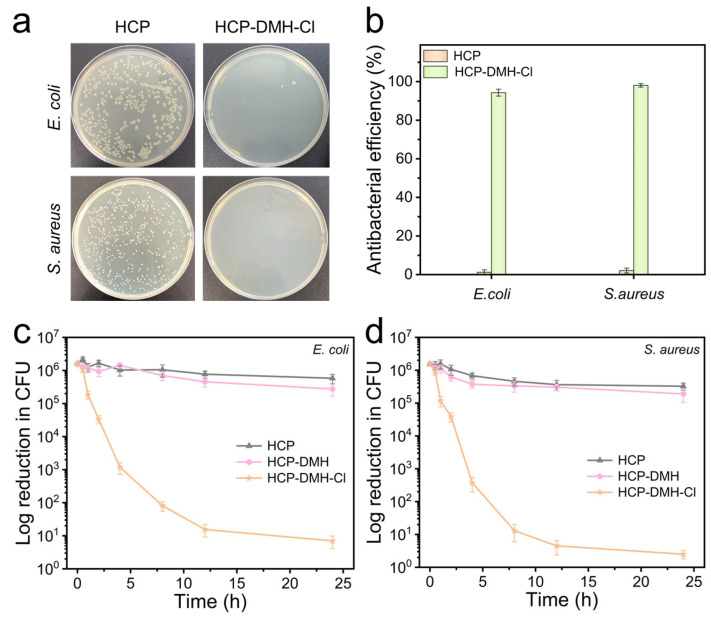
(**a**) Digital photographs of bacterial culture plates for *E. coli* and *S. aureus* treated with HCP-DMH-Cl, respectively. (**b**) Comparative antibacterial efficiency of HCP-DMH-Cl against model pathogens. Log reduction in CFU of different samples against (**c**) *E. coli* and (**d**) *S. aureus*.

The release of oxidative Cl^+^ species from *N*-halamine moieties in HCP-DMH-Cl membranes enables efficient bactericidal inactivation. To evaluate antibacterial efficacy, Gram-negative *E. coli* and Gram-positive *S. aureus* were used as model pathogens in standard plate counting assays. As shown in Figure 8a, the control group exhibited dense bacterial colonies, evident as uniform white clusters on agar plates. In contrast, HCP-DMH-Cl treatment resulted in near-complete bacterial elimination. These results demonstrate the favorable bactericidal performance of HCP-DMH-Cl, which further exhibited dose-dependent antibacterial activity. At a concentration of 200 µg/mL, HCP-DMH-Cl achieved antibacterial efficiencies of 94.5% against *E. coli* and 98.3% against *S. aureus*, respectively (Figure 8b). To further provide a more rigorous evaluation of antibacterial performance, the log reduction in viable bacterial counts was additionally determined. As shown in Figure 8c,d, HCP-DMH-Cl exhibited pronounced bactericidal activity, achieving nearly complete elimination of approximately 6 log CFU of both *E. coli* and *S. aureus*. Conversely, the corresponding reductions observed for HCP and non-chlorinated HCP-DMH were negligible under identical conditions, indicating that the strong antibacterial activity primarily originated from the chlorinated *N*-halamine functionality rather than the polymer matrix itself.

**Figure 9:**
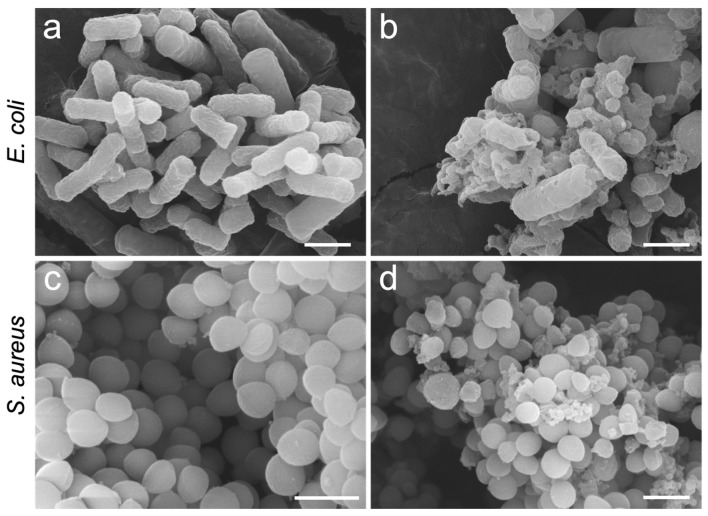
SEM images of (**a**,**b**) *E. coli* and (**c**,**d**) *S. aureus*. (**a**,**c**) represent the control groups, while (**b**,**d**) show the HCP-DMH-Cl-treated groups. Scale bars: 1 μm.

To elucidate the antibacterial mechanism of HCP-DMH-Cl, morphological alterations of bacterial cells were systematically investigated via SEM. Figure 9 displays SEM images of *E. coli* and *S. aureus* after exposure to HCP-DMH-Cl. The control group of *E. coli* exhibited a typical rod-shaped morphology with a smooth, intact cell wall (Figure 9a), whereas cells treated with HCP-DMH-Cl displayed a significantly roughened surface with irregular perforations (Figure 9b). For *S. aureus*, the control group of cells maintained a grape-like cluster structure with a homogeneous surface (Figure 9c), but treatment with HCP-DMH-Cl induced cytoplasmic leakage and cellular shrinkage (Figure 9d). These observations suggest that HCP-DMH-Cl compromises the structural integrity of the bacterial cell envelope. While the exact molecular mechanism requires further investigation, it is plausible to propose that the observed envelope disruption may be attributed to oxidative damage induced by the compound, a hypothesis consistent with the known chemistry of *N*-halamine compounds [51].

## 4. Conclusions

In this study, we report a facile synthetic strategy for preparing chloride-regenerable porous tubular antibacterial polymers via a combination of Friedel–Crafts alkylation and nucleophilic substitution reactions. DMH moieties were covalently grafted onto the tubular HCP surface through sequential bromomethylation and nucleophilic substitution modifications. During chlorination, the hydantoin groups in HCP-DMH were converted into *N*-halamine structures via in situ chlorination, yielding the bactericidal form HCP-DMH-Cl. SEM characterization revealed that the tubular morphology of HCP remained intact after multistep functionalization, demonstrating its good structural stability. Combined characterization by FT-IR, XPS, XRD, and WCA measurements confirmed the successful synthesis of HCP-DMH-Cl. The highest oxidative chlorine content in HCP-DMH-Cl was obtained at 25 °C with a chlorination time of 2 h. Antibacterial assays demonstrated that 200 µg/mL of HCP-DMH-Cl achieved sterilization efficiencies of 94.5% against *E. coli* and 98.3% against *S. aureus*. In comparison to conventional polymer-based *N*-halamines or surface-immobilized chlorinated coatings, HCP-DMH-Cl exhibits desirable stability, reusability, and diffusion properties. The polymer’s porous architecture enables intimate integration of functional groups, in sharp contrast to thin-film coatings prone to delamination or deactivation. Compared with conventional polymer-based *N*-halamines or surface-coated chlorinated materials, the present HCP-DMH-Cl system provides a porous tubular framework capable of integrating rechargeable antibacterial sites within an accessible three-dimensional architecture. This structural design may offer advantages in terms of active-site utilization, mass transport, and regeneration efficiency. Notably, the tunable halogenation degree may offer opportunities to tailor antibacterial performance for specific non-implantable applications, such as reusable antimicrobial surfaces.

## Data Availability

The data presented in this study are available upon request from the corresponding author.

## Abbreviations

The following abbreviations are used in this manuscript:

*E. coli*	*Escherichia coli*
*S. aureus*	*Staphylococcus aureus*
POPs	Porous organic polymers
HCPs	Hyper-cross-linked nanoporous polymers
BME	Bromomethyl methyl ether
DMH	5,5-Dimethylhydantoin
WCA	Water contact angle
HOCl	Hypochlorous acid
DCE	1,2-Dichloroethane
DCM	Dichloromethane
SEM	Scanning electron microscope
FT-IR	Fourier transform infrared
XPS	X-ray photoelectron spectroscopy
XRD	X-ray diffraction
